# Probing and Tuning Strain‐Localized Exciton Emission in 2D Material Bubbles at Room Temperature

**DOI:** 10.1002/adma.202503134

**Published:** 2025-09-26

**Authors:** Junze Zhou, John C. Thomas, Thomas P. Darlington, Edward S. Barnard, Atsushi Taguchi, Adam Schwartzberg, Alexander Weber‐Bargioni

**Affiliations:** ^1^ The Molecular Foundry Lawrence Berkeley National Laboratory 1 Cyclotron Road Berkeley CA 94720 USA; ^2^ Research Institute for Electronic Science Hokkaido University Sapporo Hokkaido 001‐0020 Japan

**Keywords:** 2D materials bubbles, lifetime, nanoindentation, near‐field probe, tunable emission

## Abstract

In monolayer transition metal dichalcogenides bubbles—nanoscale deformations typically exhibiting a dome‐like shape—Excitons are confined by the strain effect, which exhibits extraordinary emission properties, such as single photon generation, enhanced light emission, and spectrally tunable excitonic states. While the strain profiles of these bubbles are extensively studied, this work provides an approach 1) to directly visualize the associated exciton properties in bubbles formed in WSe_2_ monolayer, revealing an intrinsic emission wavelength shift of ≈40 nm, and 2) actively modify local strain, enabling further exciton emission tuning over a range of 50 nm. These are achieved by emission mapping and nanoindentation using a dielectric near‐field probe, which enables the detection of local emission spectra and emission lifetimes within individual bubbles. Statistical analysis of 67 bubbles uncovers an emission wavelength distribution centered around 780 nm. Furthermore, saturation behavior in the power‐dependent studies and the associated lifetime change reveal the localized nature of the strain‐induced states. These findings provide direct insights into the strain‐localized emission dynamics in bubbles and establish a robust framework for non‐destructive, reversible, and predictable nanoscale emission control, presenting a potential avenue for developing next‐generation tunable quantum optical sources.

## Graphical abstract

This work provides an approach: 1) to directly visualize the associated exciton properties, revealing an intrinsic emission wavelength shift, and 2) actively modify local strain, enabling further exciton emission tuning. These findings provide direct insights into the strain‐localized emission dynamics in bubbles and establish a robust framework for non‐destructive, reversible, and predictable nanoscale emission control, presenting a potential avenue for developing next‐generation tunable quantum optical sources.

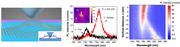

## 1 Introduction

Nanobubbles (simply referred to as “bubbles” henceforth) in 2D Transition Metal Dichalcogenides (TMDs) often form unintentionally during sample transfer or fabrication, introducing localized strain and lattice homogeneity.^[^ ^1^, ^2^ ^]^ While such features are sometimes regarded as defects, they offer a unique opportunity to engineer and enhance the optical properties of a 2D material.^[^ ^3^, ^4^ ^]^ Most intriguingly, bubbles can enable a single‐photon emitter^[^ ^5^ ^]^–a sought‐after property in direct‐bandgap 2D semiconductors that has been hindered by inhomogeneous band‐edge broadening.^[^ ^6^ ^]^ By locally modifying the band structure, bubbles create deep exciton traps that confine carriers and produce spectrally sharp, anti‐bunched photons at cryogenic temperatures.^[^ ^5^, ^7^, ^8^ ^]^ Unlike the pillar‐based approach,^[^ ^9^, ^10^, ^11^ ^]^ which induces static strain through draping monolayers over lithographically‐patterned substrate pillars, bubbles enable dynamic, in situ strain control via mechanical manipulation, such as internal or external pressure. This enables real‐time modifications to their shape, size, and strain profile, thereby tuning their emission properties.^[^ ^1^, ^12^, ^13^, ^14^ ^]^ This inherent flexibility not only distinguishes them from conventional single‐photon sources but also underscores their potential for a wide range of tunable photonic applications, such as high‐performance sensing and non‐traditional photovoltaics.

The exceptional strain sensitivity of excitons in TMDs underpins the tunability of these bubble emitters. Their high exciton binding energies^[^ ^12^ ^]^ and strong light–matter interactions enable stable photon emission,^[^ ^15^, ^16^, ^17^ ^]^ while the intrinsic elasticity of TMDs supports significant strain modulation without structural degradation.^[^ ^18^, ^19^, ^20^ ^]^ Localized strain engineering provides an effective strategy to tailor excitonic states and the electronic band structure,^[^ ^3^, ^4^, ^21^ ^]^ offering precise spatial and spectral control of quantum emitters, particularly monolayer TMD, where moderate applied tensile strain (<3%) can linearly reduce the direct bandgap using a constant gauge factor (in meV/%).^[^ ^22^ ^]^ Among various strain‐inducing methods (such as pre‐patterned substrates,^[^ ^11^, ^23^ ^]^ atomic force microscope (AFM) nanoindentation,^[^ ^24^, ^25^ ^]^ and engineered bubbles^[^ ^5^, ^26^ ^]^), naturally formed bubbles during sample stacking present unique advantages, including predictable and well‐defined strain profiles. These typically range in size from a few hundred nanometers to the micron scale.^[^ ^1^, ^5^ ^]^ According to elastic theory,^[^ ^1^, ^2^, ^27^ ^]^ the strain in bubbles is proportional to the square of the aspect ratio *h*/*r*, where *h* is the maximum height and *r* is the radius. The balance between the 2D material–substrate adhesion energy and the material's in‐plane stiffness determines the strain distribution, enabling tunable bubble emission through substrate engineering.^[^ ^4^ ^]^

Despite these advancements, key challenges remain in fully leveraging strain‐engineered bubbles for deterministic, reversible tuning of excitonic emission energies. Conventional optical microscopes lack spatial resolution and the height/strain information to uncover the emission characteristics of the submicron‐sized bubbles, whereas metallic near‐field probes significantly reduce the emission upon contact, limiting their effectiveness for precise optical measurement.^[^ ^3^, ^28^ ^]^ Addressing these challenges is critical for bridging fundamental strain‐induced band structure modulation with practical nanoscale control of light–matter interactions, including the ability to develop strain‐tunable single‐photon emitters.

In this study, we overcome these challenges by leveraging bubbles as a platform for strain engineering and employing a dielectric near‐field tip to modulate strain. Using hyperspectral photoluminescence (PL) mapping, we first resolve the spatial and spectral emission profiles of strain‐induced excitonic states, identifying a centralized wavelength distribution around 782 nm. By applying tip‐induced strain, we achieve deterministic, reversible, and linear tuning of excitonic emission wavelengths, with shifts of up to 90 nm (≈180 meV) from the unstrained exciton wavelength (energy). Furthermore, our results suggest that the localized strain acts as an exciton funnel, concentrating carriers within the strained region. Power‐dependent studies on emission intensity and lifetime corroborate this interpretation, providing novel insights into the interplay between strain and excitonic dynamics. This work establishes a robust framework for strain engineering in TMDs and highlights the potential of bubbles as tunable quantum optical sources. Our findings offer potential implications for next‐generation tunable photonic applications, particularly single‐photon emitters based on 2D materials, addressing a critical need in quantum information science.

## 2 Results

**Figure 1:**
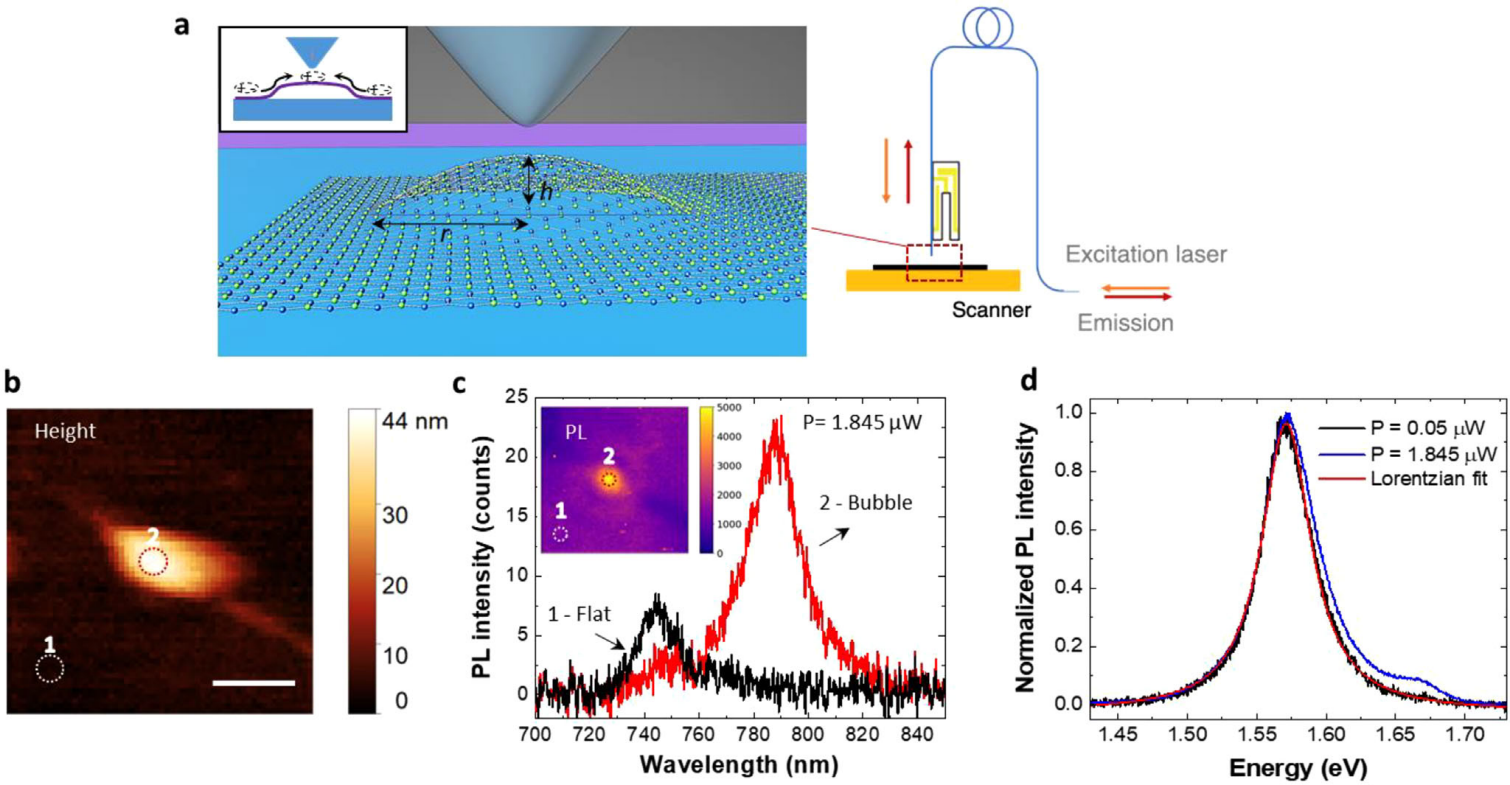
Simultaneous shear‐force height and PL emission mapping of strained bubbles in WSe_2_ monolayer with a dielectric scanning probe. a) Schematic of the scanning optical microscope setup based on a fiber probe, where the optical path is in reflection mode—the excitation laser and emission signal are all coupled through the probe. The right graph shows a close‐up view of the probe apex placed above the bubble at the interface between a WSe_2_ and h‐BN substrate. The geometry of the bubble is characterized by the height *h* and its radius *r*. The inset represents the enhanced exciton density localized at the strained bubble. b) Shear‐force height map. The scale bar is 500 nm c) PL spectra of the flat region (unstrained) and bubble site (strained) at areas 1 and 2 marked in (b). The excitation power (P) for the mapping is 1.845 µW. The inset image is the integrated PL intensity map recorded simultaneously with the one in (b). d) PL spectra recorded with different excitation powers, where the spectrum at lower excitation power (P = 0.05 µW) is fitted with a single Lorentzian peak. The *x*‐axis is converted from wavelength to energy for fitting purposes.

The bubbles are formed at the interface between the WSe_2_ monolayer and hexagonal boron nitride (h‐BN) (see Experimental section for fabrication details). To analyze the localized strain and the associated excitonic properties at the bubble site we used a dielectric near‐field probe that is capable of simultaneously recording height information and PL signal.^[^ ^29^ ^]^ During hyperspectral mappings, both excitation and collection were mediated via the fiber probe, as shown in **Figure** **1**a (see Experimental section for more details). The probe offers three key advantages: i) State‐of‐art AFM‐level topographical mapping, ii) optical spectroscopy combined with a spatial resolution near the diffraction limit (≈300 nm for light at a wavelength of 750 nm),^[^ ^29^ ^]^ suitable for bubbles larger than this dimension,^[^ ^1^, ^2^, ^4^ ^]^ iii) and the ability to perform nanoindentation without quenching effects, facilitated by the dielectric probe, enabling precise in situ monitoring of the strain‐induced excitonic emission.

By correlating the height and PL mappings on the bubbles, we analyze how the PL emission is influenced by their geometry, characterized by the height *(h*) and radius (*r*), as illustrated in the schematic view in Figure 1a. The height map in Figure 1b shows that the bubble has a height *h* of ≈40 nm and an effective width of ≈350 nm, resulting in a low aspect ratio (*h*/*r* <0.2). Despite this low aspect ratio, the excitonic properties at the bubble site are notably altered. As shown in the correlated PL map and spectra (Figure 1c), the bubble shows a threefold stronger emission peak compared to the one recorded from the flat region, accompanied by a wavelength shift of ≈40 nm. This enhanced emission rate at the bubble site has been attributed to increased confinement in the strain‐induced potential well, which improves the oscillator strength of excitons.^[^ ^30^ ^]^ The localized strain can be extracted from the emission energy shift, which is ≈0.9%. This is derived from the relation Δ*E* = αε, where α is a constant representing the energy shift (Δ*E*) per unit strain (ε), which is ≈100 meV per percent of strain for the WSe_2_ monolayer.^[^ ^14^, ^31^, ^32^ ^]^ This strain estimate aligns with the calculated strain using the Föppl–von Kármán (FvK) equations (See Figure S1, Supporting Information),^[^ ^27^ ^]^ which indicate a maximum strain of ≈1% at the bubble, decreasing smoothly toward the edge.

During the mapping experiment, an excitation power of 1.845 µW is used to ensure a good signal‐to‐noise ratio. Under this power, a small peak near the free exciton wavelength is observed alongside the strain‐localized peak (Figure 1c). However, this small peak disappears when excitation power is lowered to 0.05 µW as shown in Figure 1d, suggesting that strain‐localized states are reaching their maximum occupancy under the high power excitation, resulting in the emission of free excitonic states. This phenomenon is analogous to the saturation of defect‐related emission in ZnO where, as the photoexcitation fluence and exciton density exceed the number density of the defect‐related states, the band‐edge emission increases.^[^ ^33^, ^34^ ^]^ Further evidence of the saturation effect, referring to the condition where increasing excitation power no longer leads to a corresponding increase in the emission intensity due to the finite occupancy of the excitonic states, will be provided by power‐dependence measurements in the following section. Notably, the strain‐localized peak can be well fitted with a Lorentzian peak. This can be attributed to the reduced exciton‐phonon coupling^[^ ^35^ ^]^ and the exciton funneling effect.^[^ ^36^ ^]^ In the former case, the decrease in exciton–photon interactions allows more excitons to remain within the light cone, enhancing the quantum yield of the associated excitonic emission.^[^ ^35^ ^]^ In the latter case, the strain gradient in our ≈350 nm diameter bubble, as evidenced in the strain map (Figure S1, Supporting Information), can promote exciton migrating, or funneling, toward the lowest energy potential leading to a dominant excitonic emission from the bubble apex where the energy minimum of the potential well is localized. Therefore, even though the dielectric probe collects the emission signal from an area of ≈300 nm,^[^ ^29^ ^]^ which is comparable to the dimension of the bubble and larger than the area of maximum strain at bubble apex, the dominant emission from the bubble apex indicates an efficient funneling effect.

**Figure 2:**
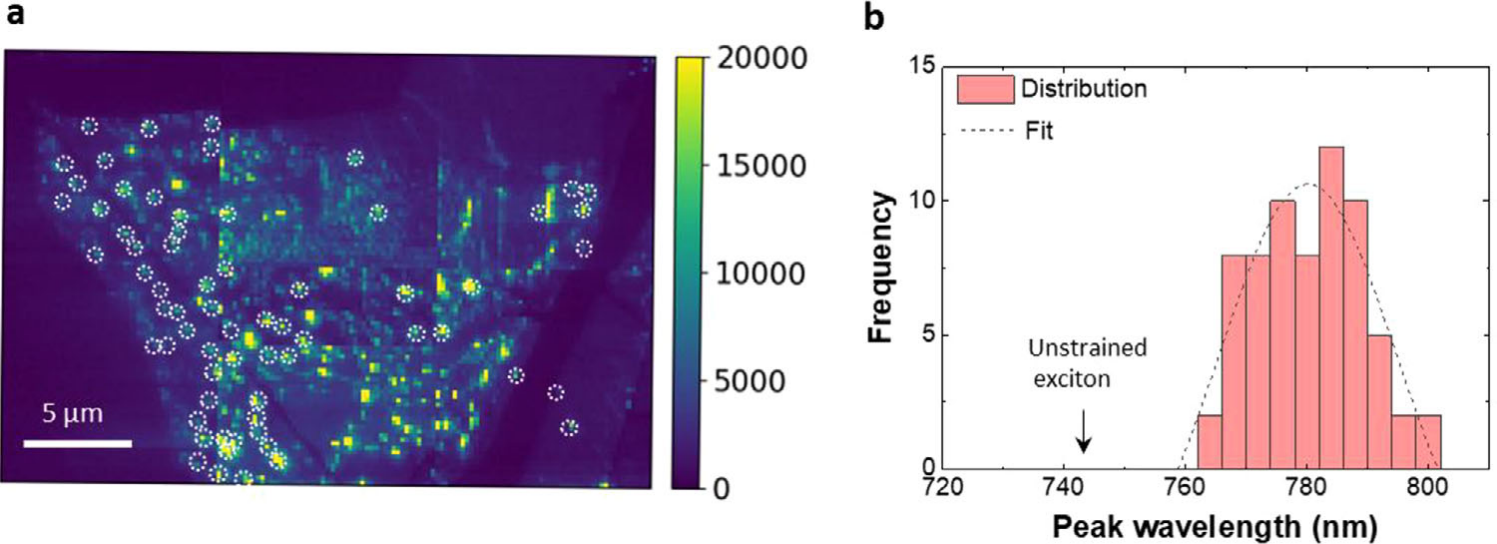
Universal PL characteristics within the sample. a) PL emission map across an area of 20 × 30 µm^2^. b) Histogram of peak wavelength positions of the emission spectra from the 67 individual bubbles marked by the white circles. The distribution is fitted with a Gaussian curve.

To statistically analyze the peak shift of bubble emissions in the sample stacked on h‐BN, we perform large‐scale hyperspectral scans. **Figure** **2**a presents a 20 × 30 µm^2^ area PL map, composited from six separate scans acquired using the same probe. The light‐emitting regions, with signal integrated over 700–850 nm at each pixel, correspond to TMD‐covered areas, featuring luminescent hotspots from randomly distributed bubble formations (see Figure 2.1a in the Supporting Information for height scans). The peak wavelengths of 67 bubbles (marked by the white circles) are analyzed, and Figure 2b presents the histogram of their wavelength positions. The shifted wavelengths range from 760 to 800 nm and follow a Gaussian distribution centered at around 782 nm, corresponding to an 80 meV energy shift from the unstrained free excitonic energy.

To further understand the origin of the PL peak shift, we examine the geometric characteristics of the bubbles, particularly their aspect ratio (*h*/*r*), which is a critical parameter influencing their strain profiles. Statistical analysis of the height map (See Figure S2.1b in the Supporting Information) reveals that the aspect ratio distribution is centered around 0.12, consistent with previous studies on the bubbles formed at the WSe_2_–h‐BN interface.^[^ ^37^ ^]^ This is further confirmed by the analysis of a larger number of bubbles (See Figure S2.1c in the Supporting Information). While the central wavelength shift aligns with the central aspect ratio distribution, exceptional cases – such as the bubble with pyramidal shape and sharp features, shown in Figure S3a (Supporting Information), deviate from universal scaling due to their irregular geometry.^[^ ^1^ ^]^ The bubble in Figure S3a (Supporting Information) exhibits a larger aspect ratio with greater deformation of the 2D material, suggesting a higher overall strain, resulting in an emission peak at a larger wavelength shift (Figure S3b, Supporting Information). This deviation is confirmed by the linear trench between the emission energy shift and aspect ratio squared, when we removed those exhibiting less rounded geometries (see Figure S2.2, Supporting Information). Nevertheless, the general strain characteristics of the bubbles, governed by the material properties and substrate adhesion, impose a limit on their emission tunability.

**Figure 3:**
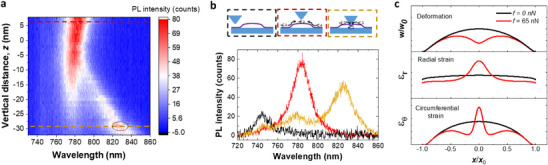
Evolution of PL spectra under nanoindentation. a) PL spectra recorded as stage position changes from −32 to 8 nm (the initial scanning probe–sample distance). b) Emission spectra recorded in three different cases: Tip on the flat region, tip on the bubble at *z* = 6 nm as marked by the red dashed line in a, and tip on the intended bubble at *z* = −29 nm as marked by the yellow dashed line in a. c) Calculated deformation and strain profiles based on the nonlinear plate theory (see Supporting Information for details), where out‐of‐plane deformation *w*, in‐plane radial strain ε_*r*_, and circumferential strain ε_θ_ are calculated with and without nanoindentation. A tip‐loading force *f* = 65 nN is applied at the center position of the probe.

To overcome the intrinsic limitations in the emission tunability of 2D bubbles, we utilize tip‐induced strain to achieve deterministic control of the emission wavelength through localized nanoindentation. By performing nanoindentation and monitoring in situ PL changes with the dielectric probe, we observe significant modifications in the emission spectrum, as illustrated in **Figure** **3**a. The evolution of the PL emission unfolds in three sequential stages. In the initial stage, as the tip approaches the bubble but does not make contact, the emission remains unchanged when the tip position changes from 8 to 0 nm. In the second stage, between 0 and ≈−7 nm, the emission wavelength changes from 783 to 778 nm, where the initial wavelength is associated with in‐plane tensile strain induced by the formation of the bubble. The slight blue shift in the emission is observed, indicating a reduction in strain. This shift can be attributed to a transition where the bubble's internal pressure is counteracted by the tip‐induced pressure, causing the flattening of the outward bending of the bubble due to the tip pressure. The energy and intensity changes in this stage are minor, indicating that the applied strain is localized to a small contact region directly under the probe (where *r*_tip_ << *r*_bubble_), while the overall bubble shape remains intact. This localized interaction aligns with the actual size of the probe^[^ ^29^ ^]^ and nanoindentation experiments in the literature.^[^ ^1^ ^]^ In the third stage, as the tip continues to indent the bubble, the emission spectrum exhibits “peak splitting,” with the lowest energy peak undergoing a redshift. The maximum wavelength shift reaches ≈832 nm, with an additional spectral shift of about 50 nm induced by the probe compared to the bubble's emission, highlighting the significant impact of tip‐induced strain on the bubble's excitonic properties.

In Figure 3b, we compare the spectra recorded in different scenarios: flat region, bubble, and indented bubble. In addition to the indentation‐induced emission peak, two extra peaks are observed, aligning with the emission positions at the free exciton and bubble emission energies. The appearance of the free exciton peak can be attributed to the saturation of the localized states, as an excitation power of 1.845 µW is used—similar to hyperspectral mapping—to minimize the stage drift while ensuring a good signal‐to‐noise ratio. The coexistence of the bubble emission peak highlights the localized nature of the nanoindentation, confined to the small area directly under the probe.^[^ ^1^ ^]^ Notably, the majority of excitons emit from the lowest energy, which shifts as a function of the tip‐induced strain. This enhanced emission is attributed to an additional exciton funneling channel created by the tip, which represents the lowest energy state. Consequently, excitons preferentially populate this lowest energy state, as illustrated in the schematics. Both the enhanced emission due to exciton funneling and the magnitude of PL shift can be attributed to the additional localized strain induced by the tip apex, as modeled in Figure 3c, based on the tip geometry measured via SEM (see Note S5 in Supporting Information). Analysis of the strain profile using nonlinear plate theory, originally developed for localized strain in graphene,^[^ ^2^, ^38^ ^]^ shows that the in‐plane components of the strain are sufficient to reproduce the experimentally observed PL shift (see Note S6 in Supporting Information for calculation details). This is due to the atomically thin nature of the 2D material makes the in‐plane strain effects dominate the mechanical response. When the indentation force is applied to the bubble region, it causes inward bending of the membrane (see Figure 3c), producing a strong strain gradient of the radial and circumferential strain, with the maximum strain located directly under the tip. In addition to the wavelength position shift, we also observe a local intensity maximum at the position *z* = −29 nm as marked by the black dotted circle in Figure 3a. This suggests that the emission intensity does not have a monochromatic relation with the strain value. It may be influenced by the hybridization of bent excitonic states with defect states.^[^ ^14^, ^39^ ^]^ We are excluding the strain effect on the dark states, as our present measurements are performed at room temperature, where the PL spectrum is dominated by the bright exciton emission. Under these conditions, the dark exciton feature is spectrally unresolved without external Purcell enhancement due to its intrinsically weaker oscillator strength and its overlap with the dominant bright exciton peak.^[^ ^40^ ^]^

**Figure 4:**
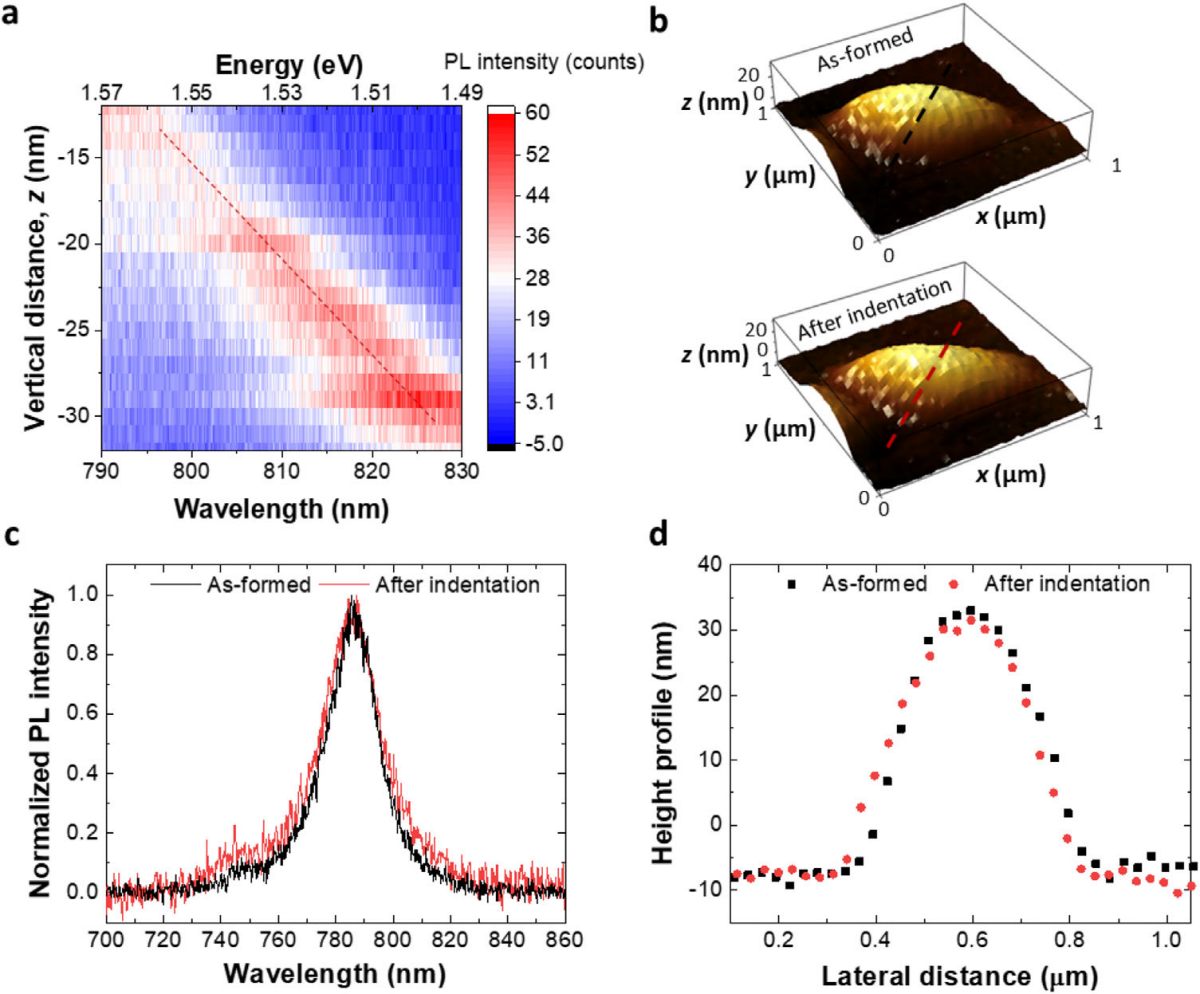
Elastic deformation induced by the tip. a) Linear evolution of the emission wavelength as a function of nanoindentation depth. b–d) Topography, emission spectra, and height profiles of the bubble before and after the nanoindentation.

**Figure 5:**
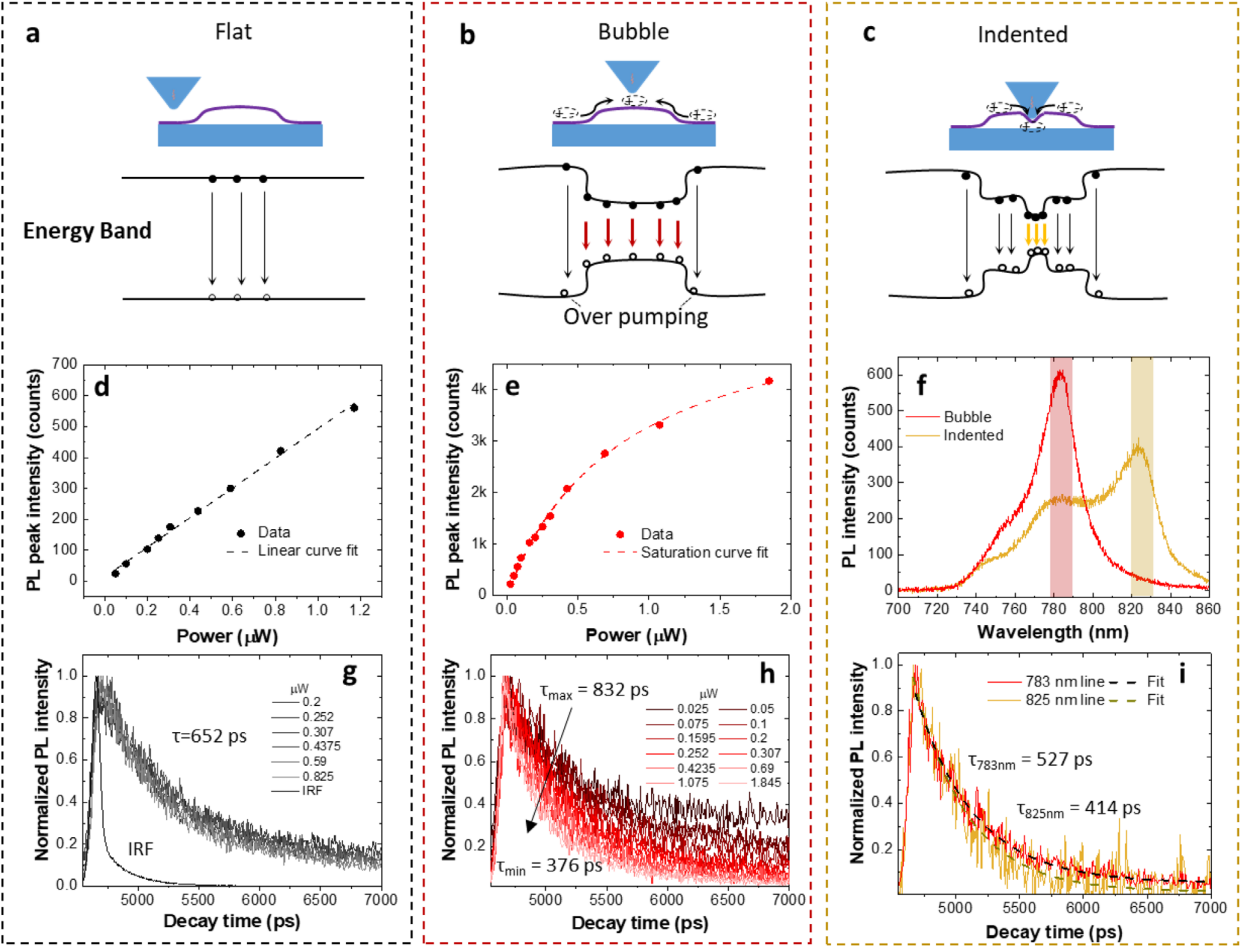
Photophysics of the strain‐induced localized states. a–c) Energy band diagrams and proposed mechanism of the electron transitions in three different cases: flat region, bubble with built‐in strain, and bubble under tip‐induced strain. d,e) Power‐dependent PL intensity measured on the flat region and bubble, and the fittings to a linear curve and a saturation curve, respectively. g,h) PL intensity decay as a function of excitation power measured on the flat region and bubble with the filtered peak at 743 and 780 nm, respectively. f) PL spectra recorded when the tuning fork feedback is set at the setpoints of 95% and 64%. i) PL intensity decays of the bubble emission and the tip‐strain‐induced peaks (wavelength ranges outlined by red and yellow regions in (f). The excitation power is 0.4235 µW.

In the zoomed‐in view of PL evolution (Figure **4**a), we observe a linear wavelength shift as a function of tip–sample distance. This reproducible linearity (see extra data in Figure S4 in the Supporting Information for the reproducibility) can be interpreted using the small nanoindentation approximation, where the strain is proportional to *δ*/*r*, with *δ* representing the indentation depth and *r* the bubble radius. This approximation is valid in the elastic deformation regime, where the materials’ response is proportional to the applied force (*F*). The linearity of the wavelength shift implies that the tunability is not only controllable but also predictable by adjusting the indentation depth through the vertical movement of the sample stage. Additionally, since *F* is proportional to the amplitude damping of the tuning fork oscillation, the indentation depth can also be controlled by fine‐tuning the tuning fork setpoint, which in turn adjusts the tip‐induced strain. This approach is applied in the following section to obtain static PL spectra under tip strain (Figure 5f). Such precise control is invaluable for practical applications where specific emission wavelengths are required.

The elastic nature of this response is further confirmed by the unchanged bubble shape before and after the nanoindentation, as shown in Figure 4b,d. The stability of the bubble's geometry under repeated indentations indicates that the material remains within its elastic limit, avoiding any plastic or permanent deformation. As a result, the intrinsic emission of the bubble remains consistent, and the strain‐induced shifts in the emission wavelength are fully reversible when the tip is retracted, as demonstrated by the overlapped PL spectra before and after indentation in Figure 4c. This ability to non‐destructively induce, linearly control, and reversibly tune localized emission is essential for practical applications such as strain‐induced quantum emitters.

## 3 Discussion

The associated electronic transitions for excitonic emission in the three different scenarios (flat region, bubble, and indented bubble) are proposed schematically in the energy band diagrams shown in **Figure** **5**a–c. Compared to the cases where the probe scans over the flat region or unindented bubbles, nanoindentation introduces an additional localized state directly under the tip. This state enables excitons to be trapped and guided into the lowest energy configuration within the indented region. As previously discussed, high excitation power is required in both hyperspectral scans and nanoindentation experiments to minimize stage drift. Under these conditions, the strain‐localized states reach saturation, where exciton density exceeds the maximum capacity, preventing additional excitons from remaining confined to the lowest energy state. This saturation results in the appearance of both free exciton and bubble emission peaks in the nanoindented PL spectrum.

To validate the proposed mechanism, we investigate the exciton dynamics in the localized state by conducting power‐dependent studies and analyzing the associated lifetime variations. The PL lifetimes were extracted by fitting the time‐resolved PL data using a single‐exponential decay model, *I*(*t*) = *I*_0_ *exp*(− *t*/τ_PL_), where *I*(*t*) is the intensity as a function of time, *I*_0_ is the initial intensity, and τ_PL_ is the PL lifetime. This fitting approach is applied consistently across all measured regions to ensure comparability. The fitting results reveal distinct differences between free exciton and bubble emission. As shown in Figure 5d,g, the free exciton emission measured on the flat region remains in the linear region, with PL intensity increasing linearly with excitation power. The corresponding lifetime remains constant at ≈652 ps, indicating minimal power dependence. In contrast, the bubble emission exhibits clear signs of saturation with increasing excitation power (Figure 5e), described by the fit *I* = *I*_sat_ *P*/(*P* + *P*_Sat_), where *I*_sat_ is the saturated emission intensity and *P*_Sat_ is the saturation power. The fitted *P*_Sat_ is 0.82 µW, representing a low excitation power. Concurrently, the lifetime of bubble emission decreases from 832 to 376 ps with increasing power (Figure 5h).

We propose that the observed decrease in lifetime is mainly due to the increase in non‐radiative recombination at the physically confined strain‐localized state. At low excitation power, excitons preferentially recombine at these strain‐localized sites, resulting in a single peak within increased symmetry (Figure 1d). As the excitation increases, the PL lifetime decreases. This behavior occurs because the photoexcited carrier in the confined 2D exciton reservoir saturates at the bubble site, as evidenced by the intensity saturation. This saturation enhances the likelihood of non‐radiative processes such as auger recombination^[^ ^41^, ^42^ ^]^ and exciton–exciton coulomb interactions,^[^ ^43^, ^44^, ^45^ ^]^ indicated by the slight linewidth broadening and blueshift of the emission peak at the higher excitation power (see Figure S7, Supporting Information). These processes introduce additional decay pathways that compete with radiative recombination, thereby shortening the τ_PL_.^[^ Notably, we have deterministically shown the longer lifetime of strain‐localized states using our probe, which aligns with previous reports of bubble‐related single photon emissions at low temperatures, where lifetimes typically reach the nanosecond range.^[^ ^9^, ^46^, ^47^, ^48^ ^]^ However, it is not possible to lower excitation power further to explore this behavior more deeply in our current setup due to detection limits at ambient conditions.

Building on these findings, we measure the lifetime of the tip‐strain‐induced emission line by reducing the tuning fork's free amplitude setpoint from 95% to 64%. The maximum PL emission shifts by around 43 nm as shown in Figure 5f. The lifetime measurement is performed under an excitation power of 0.4235 µW with a 1‐s integration time. The measured lifetime of the 825 nm line is 414 ps, shorter than that of the un‐indented bubble emission line (527 ps), as shown in Figure 5i. This shorter lifetime indicates faster exciton dynamics in the tip‐strain‐induced state, likely due to the increased localization and lower exciton occupancy density within the more physically confined strained area. Additionally, we note that, although the indenter is purely dielectric, the higher refractive index (n ≈ 1.5)^[^ ^49^ ^]^ increases the local density of photonic states, which can enhance radiative recombination and further influence the measured lifetime. Due to instrumental limitations, we are unable to perform a stable power‐dependent study under this configuration. Nevertheless, our analysis of bubble strain emission strongly suggests that the tip‐induced state also reaches saturation under high excitation conditions.

To contextualize these findings, we present schematic diagrams illustrating the mechanisms of excitonic transitions observed in static PL spectra in Figure 3b. For the bubble emission spectrum, the strain‐localized state accounts for the main peak, while the free exciton peak arises due to the saturation, as illustrated in Figure 5b. Similarly, in the tip‐strain scenario, the emission spectrum comprises three distinct transitions. Among these, the transition associated with the tip‐strain state dominates, as it represents the lowest energy state, as illustrated in Figure 5c. These observations highlight the interplay between localized states and free excitons under varying strain conditions and demonstrate the tunability of excitonic emission in 2D materials controlled through the tip strain.

## 4 Conclusion

In summary, we investigate the excitonic properties induced by the built‐in strain in 2D material bubbles and demonstrate their tunability using a dielectric near‐field probe. By leveraging the capability of our probe to perform simultaneous topographic and spectroscopic measurements, we achieved a high‐resolution spectral readout at the bubble sites. This allows us to reveal a centralized peak shift of ≈80 meV, determined by the intrinsic properties of the substrate and 2D material. Furthermore, we demonstrate a reversible and precise linear tunability of strain‐localized states through tip nanoindentation, achieving an energy shift of up to 180 meV as the tip‐strain further reduces the bandgap energy. This exceeds the tunability previously reported in modulating the strain‐localized emission at wrinkle sites.^[^ ^50^ ^]^ Our investigations into the exciton dynamics reveal that exciton emission at the strained bubble exhibits a longer lifetime compared to free exciton states. The saturation of these states at low excitation power highlights their efficiency in excitonic trapping and recombination processes. Unlike prior methods, which often suffered from spatial mismatch or introduced charging effects, our approach provides a robust, non‐destructive, and repeatable means to explore strain‐tunable excitonic properties with nanoscale precision. These results highlight the potential of strain engineering as a powerful tool for enabling tuning excitonic properties in 2D materials.  Our findings also align with the ongoing efforts to create bubbles in a more controlled manner, such as through bulging techniques^[^ ^51^ ^]^ and proton irradiation methods.^[^ ^52^ ^]^ which may facilitate the on‐chip integration of optical cavity and waveguide for effective light coupling. Moreover, integrating our dielectric probe‐based technique with other platforms such as a cryogenic chamber could further broaden its applicability, paving the way for the development of next‐generation quantum emitters.

## 5 Experimental Section

### Sample Preparation

The WSe_2_ monolayer and hexagonal boron nitride (h‐BN) were mechanically exfoliated. Bubbles were created by mechanically stacking the WSe_2_ monolayer on h‐BN. During this process, bubbles naturally form as interfacial contaminants (primarily water and hydrocarbons) were trapped and compressed by the strong adhesive forces between 2D materials and the substrate.^[^ ^11^, ^25^ ^]^ In the conventional transfer method, h‐BN was exfoliated and transferred to a substrate first, followed by exfoliating and transferring the TMD monolayer onto the h‐BN‐coated substrate. To promote the formation of uniformly shaped bubbles, this approach was modified by directly exfoliating the h‐BN onto the substrate before transferring the TMD monolayer. This ensures a cleaner and flatter surface, supporting consistent adhesion.

### The Nanoimprinted Near‐Field Fiber Probe

1) A cleaved single‐mode fiber (630HP, Thorlabs) was dip‐coated with Ormocomp photoresist. 2) The coated fiber was mounted onto a piezo stage above the transparent mold featuring an inverted pyramidal structure. 3) The fiber core was located by coupling red light through the fiber, and the transmitted light was monitored via a camera. This enabled precise alignment of the fiber core with the center of the mold. 4) UV light was illuminated from below the mold to expose and cure the Ormocomp for ≈3 min, solidifying the pyramid structure. 5) The mold was carefully removed, leaving a well‐defined pyramid shape integrated onto the fiber facet, with the pyramid's base covering the fiber core. The nanoimprinted pyramid probe has a tip curvature of 20 – 30 nm (See SEM images in Note S5 of the Supporting Information).

### Steady‐State PL and Time‐Resolved PL Measurement

The PL measurements were carried out in a fiber‐in–fiber‐out configuration. In this setup, a 633 nm excitation laser (FWHM = 6 ps, repetition rate of 60 MHz), derived from the Supercontinuum laser source (Leukos LMS 7026), was modulated using an acoustic‐optics modulator and coupled to the fiber using the Thorlabs MBT612D fiber launch stage with a 10× objective lens (0.25 NA). The emitted signal from beneath the probe was collected through the same path and directed to the spectrometer (Andor Kymera 328i), which recorded a spectrum ranging from 624 to 913 nm. The lifetime was measured using a single photon avalanche diode (PicoQuant PDM series) and a single photon counting module (HydraHarp 400). The time resolution of this setup is ≈60 ps.

### Nanoimaging and Nanoindentation

The scanning near‐field optical microscope is based on the Horiba CombiScope microscope equipped with a shear‐force head. The near‐field probe was attached to a tuning fork oscillating at around 35 kHz, which modulated the tip–sample distance. Typically, the tuning fork oscillation amplitude was set to 95% of its free amplitude, maintaining a constant tip‐to‐sample distance. During scanning, the tip recorded both the shear‐force height information and the emission spectra simultaneously at each pixel. A typical integration time of 0.1 s per pixel was used for PL data acquisition. Two approaches were employed in the nanoindentation experiments: 1) The spectra stack function in the Horiba software was used to capture sequential PL spectra while varying the stage height incrementally with a step size of 1 nm and an integration time of 0.1 s. 2) The setpoint of the tuning fork was reduced to achieve a fixed indentation depth, allowing for the acquisition of spectra with a longer integration time.

## Conflict of Interest

The authors declare no conflict of interest.

## Supporting information



Supporting Information

## Data Availability

The data that support the findings of this study are available from the corresponding author upon reasonable request.
