# Supplementary material for: Probing and Tuning Strain‐Localized Exciton Emission in 2D Material Bubbles at Room Temperature
Source: Adv Mater. 2025 Sep 26;38(3):e03134. doi: 10.1002/adma.202503134 (PMC12801371; doi:10.1002/adma.202503134)
Supplement: Supplementary file 1 — Supporting Information [file ADMA-38-e03134-s001.docx]

Supplementary information for

**Probing and Tuning Strain-localized Exciton Emission in 2D Material Bubbles at Room Temperature**

*Junze Zhou^1*^*, *John C. Thomas^1^*, *Thomas P. Darlington^1*^*, *Edward S. Barnard^1^*, *Atsushi Taguchi^2^*, *Adam Schwartzberg^1^*, *Alexander Weber-Bargioni^1*^*

*^1^The Molecular Foundry, Lawrence Berkeley National Laboratory, 1 Cyclotron Road, Berkeley, California 94720, USA*

*^2^Research Institute for Electronic Science, Hokkaido University, Sapporo, Hokkaido 001-0020, Japan*

** Corresponding authors: junzezhou@lbl.gov*, tpdarlington@lbl.gov, *afweber-bargioni@lbl.gov*

# Note S1: Strain map calculation

To estimate the strain in the nanobubble we used the numerical approach developed by Darlington, et al. [*J. Chem. Phys.* **153**, 024702 (2020); *Nat Commun* **15**, 1543 (2024)]. The model assumes a Föppl–von Kármán plate for the 2D layer subjected to normal load. With the height known, the problem reduces to a linear, inhomogeneous biharmonic equation with the local Gaussian curvature as the source term. The equation is solved numerically in python using the open source finite element method library SfePy [*Adv Comput Math* **45**, 1897–1921 (2019)], using a regular triangular mesh, triangulated from a $60 \times60$ parent regular grid. To estimate the Gaussian curvature, a topography in Fig 1b was first upsampled to $128 \times128$. A user-defined local spatial filter was then applied to match the assumed boundary conditions of zero *z*-topography at the plate edge, followed by low-pass filtering to remove high frequency noise rippling from the sharp cutoff of the spatial filter. The Gaussian curvature was then calculated by numerical differentiation via finite differences. The resulting strain gradient follows the tomography of the bubble, with the maximum strain of approximately 1% located at the bubble apex. This calculated strain is close to the 0.9% that we estimated from the optical measurements by applying the gauge factor (100 meV/%)


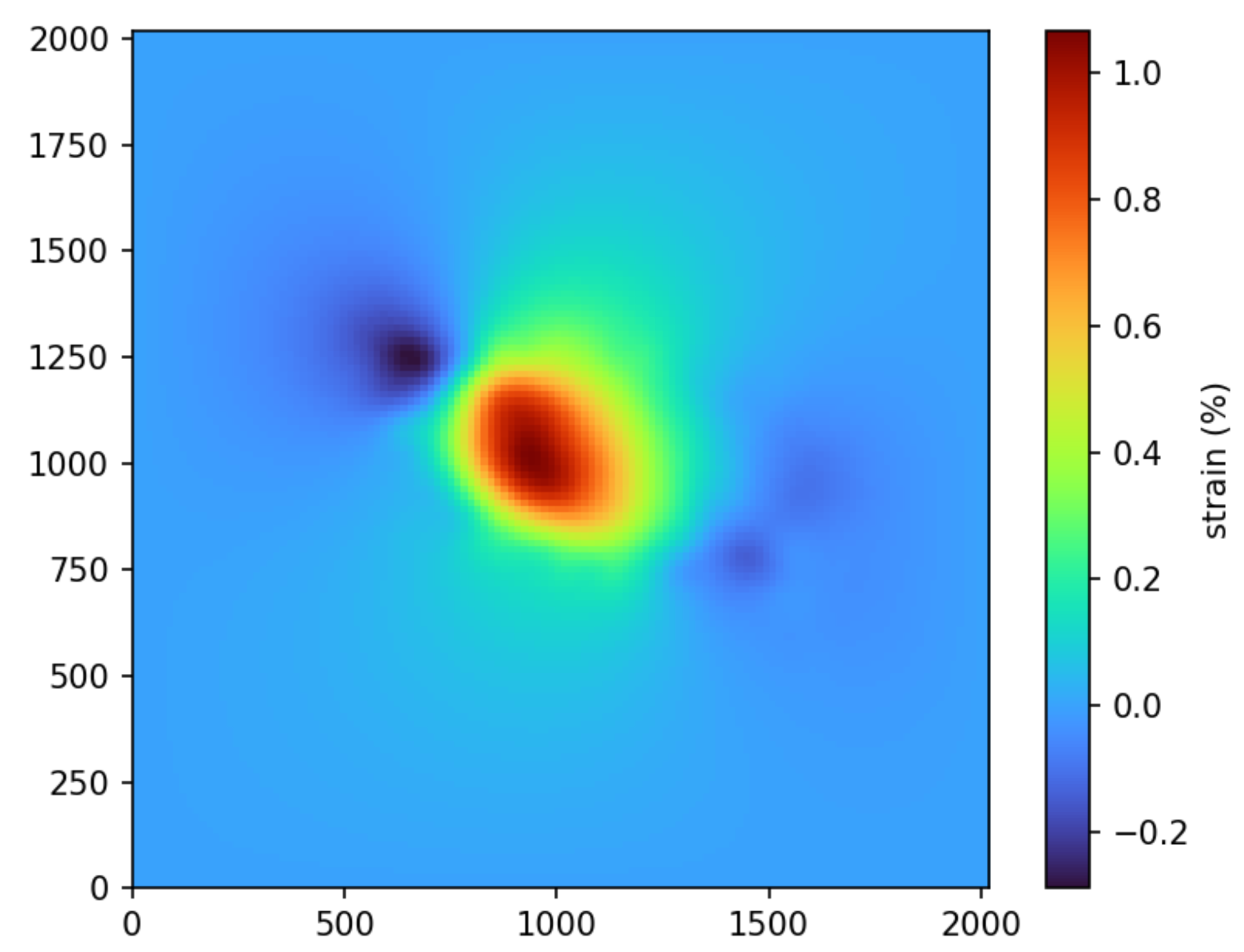


Fig. S1: Strain map calculated from the height profile in Fig. 1b.

# Note S2: Topographical analysis of bubble aspect ratios

The 67 bubbles in **Fig. S2.1** were chosen based on their enhanced and shifted emission peaks. These bubbles within our 2D materials–substrate combination can take on various shapes, including round, triangular, and pyramidal forms with sharp features, which lead to deviations from the universal scaling. To address these deviations, we re-examined the shape of 67 bubbles and removed those exhibiting less rounded geometries. We then replotted the curve using the remaining 23 bubbles (see topography image in **Fig. S2.2**). As shown in **Fig. S2.2b**, a linear fit to this subset yielded a slope value of -1.61. We use this slope to calculate the emission energy shift of the bubble studied in the paper (**Fig. 1**), where the aspect ratio squared is 0.0522, resulting in an energy shift of 84 meV. This value aligns well with the energy shift we find experimentally in the PL spectrum shift of ~90 meV. Therefore, these results support the validity of the strain-localization model in our study.


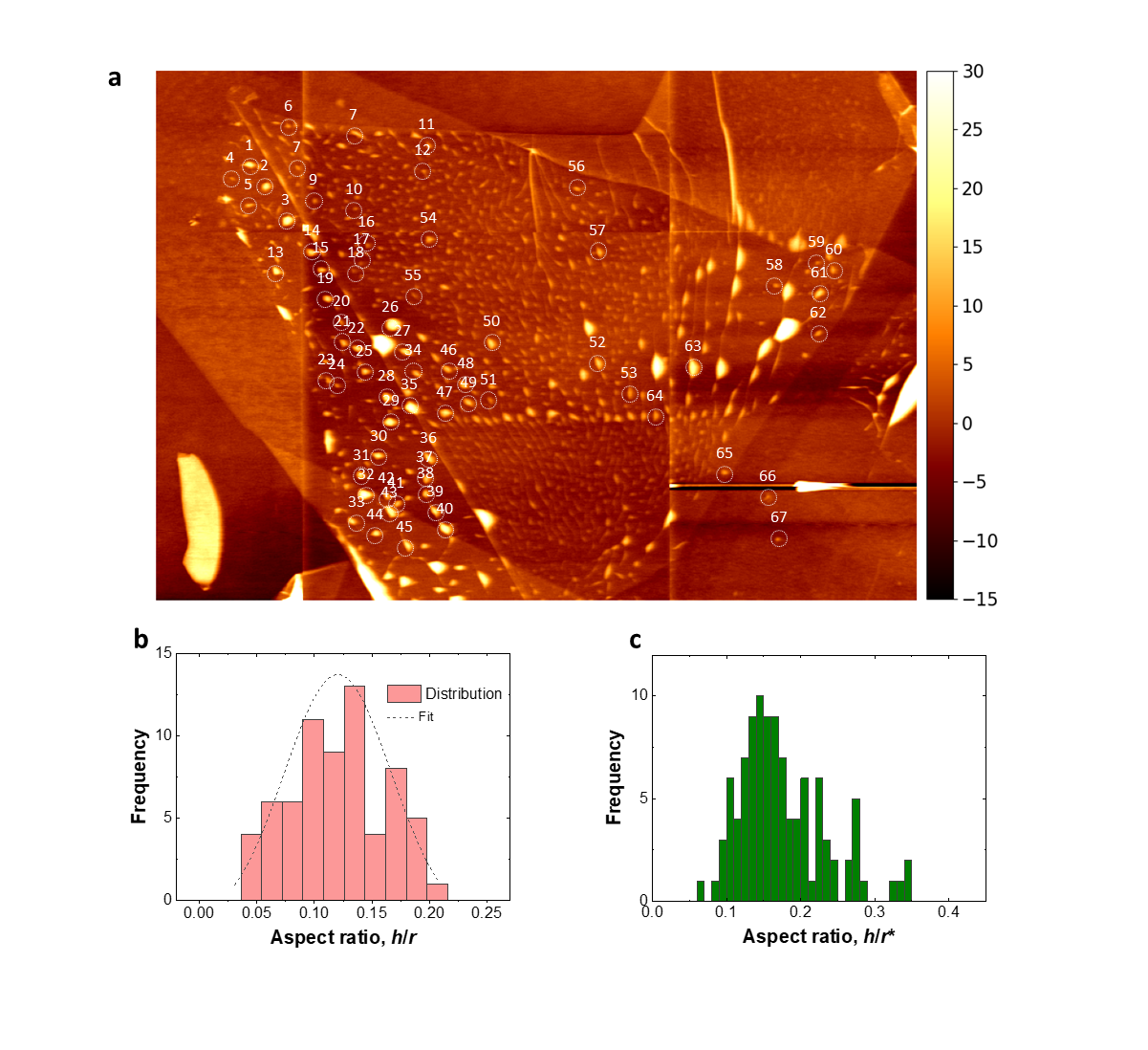


Fig. S2.1: Topographical analysis of bubbles. (a) AFM image captured using a cantilever-type AFM probe (ATEC-NC) before the near-field measurement. The labeled bubbles correspond to the positions marked in Fig. 2a. (b) Distribution of aspect ratios (*h*/*r*) for the labeled bubbles, where *h* and *r* are extracted from the AFM height profile. (c) Distribution of the aspect ratios (*h*/*r^*^*) for 104 bubbles selected using ImageJ software with the condition *h* > 5 nm. The effective radius *r*^*^ is determined as $\sqrt{A/\pi}$, where *A* is the measured area of the base of the bubble.


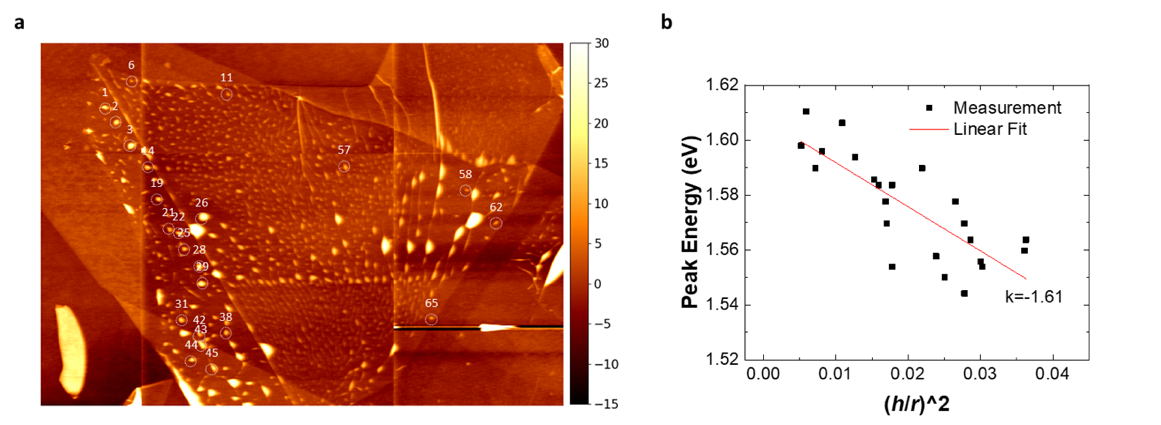


Fig. S2.2: (a) AFM image captured using a cantilever-type AFM probe (ATEC-NC) before the near-field measurement. The labeled bubbles correspond to the selected bubbles, removing exhibiting less rounded geometries. (b) Plot of emission peak energy versus aspect ratio squared, for the 23 bubbles.

**Note S3:**


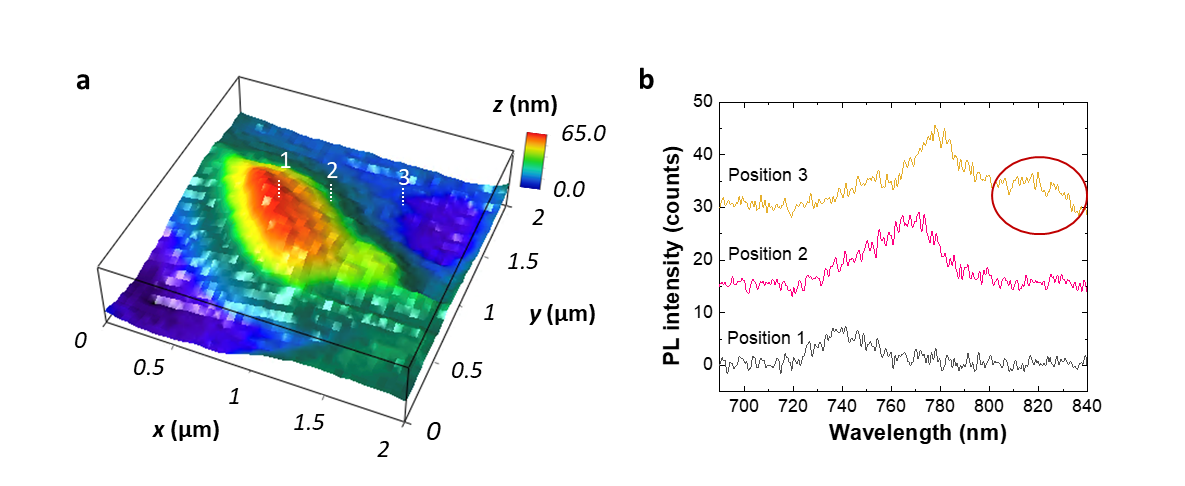


Fig. S3: Height map and spectral analysis of a tent-shaped bubble captured using the fiber probe. (a) 3D shear-force height map. (b) Corresponding PL spectra recorded in the positions 1, 2, and 3 as marked in (a). The spectrum at the top of the bubble (position 3) exhibits a peak at 820 nm, highlighted by the red circle.

**Note S4:**


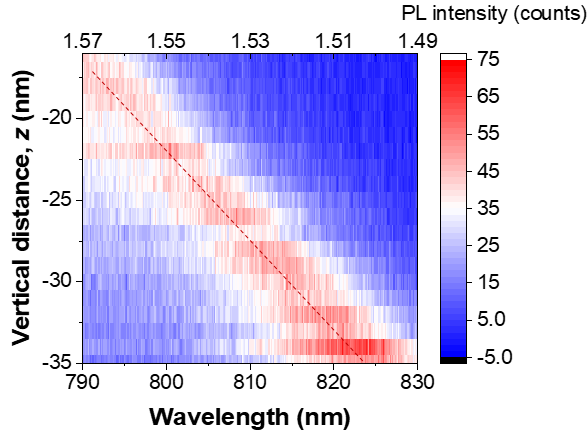


Fig. S4: Repeated measurement of the linear evolution of the emission wavelength as a function of nanoindentation depth.

**Note S5:**


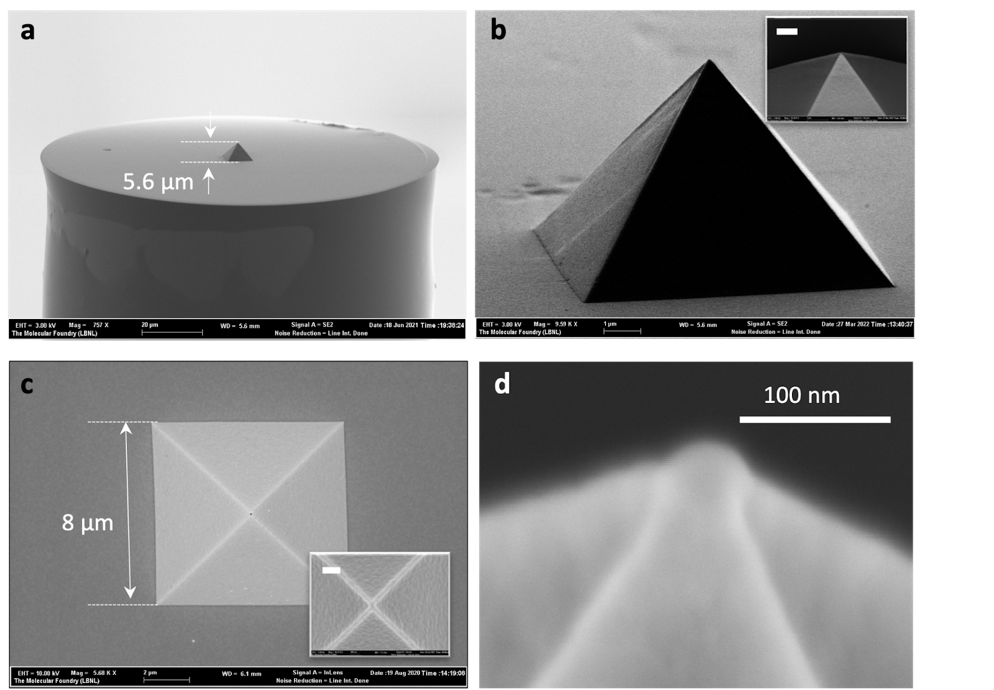


Fig. S5: SEM images of the nanoimprinted dielectric probe with a height of 5.6 μm and a base of 8 μm. (a) Tilted view of the pyramid tip on the facet of a fiber, showcasing its overall structure. (b) Tilted view of the pyramid with a zoomed-in view of the apex, featuring a scalebar of 1μm. (c) Top view of the pyramid with a zoomed-in view of the apex, featuring a scalebar of 200 nm. (d) Tilted view of the tip apex, illustrating the curvature of the tip with an estimated size of 20 – 30 nm.

**Note S6: Simulation on Tip indentation**

We calculated the deformation profile of a bubble under tip strain using the nonlinear plate theory developed in [*J. Appl. Mechanics*, **80**, 040905, (2013)][Phys. Rev. Lett., **121**, 266101, 2018]. The governing equations for the deformation of circular 2D material bubbles are derived from the axisymmetric form of Föppl-von Kármán (FvK) equations. The in-plane equilibrium equation is given by:

$$\frac{d^{2}u}{dr^{2}}+\frac{1}{r}\frac{du}{dr}-\frac{u}{r^{2}}=-\frac{1-v}{2r}{(\frac{dw}{dr})}^{2}-\frac{dw}{dr}\frac{d^{2}w}{dr^{2}}$$

Where $u$ is the in-plane radial displacement (the circumferential displacement is assumed to be zero due to symmetry), $r$ is the radius of the circular bubble, $v$ is the Poisson ratio and $w$ is the lateral deflection. This equation balances the radial stretching of the membrane with the geometric nonlinearities induced by out-of-plane deflection. The Poisson ratio *v* serves as a coupling parameter between radial and circumferential strains.

The out-of-plane equilibrium equation is given by:

$$D(\frac{d^{3}w}{dr^{3}}+\frac{1}{r}\frac{d^{2}w}{dr^{2}}-\frac{1}{r^{2}}\frac{dw}{dr})-\frac{E_{2D}}{1-v^{2}}\frac{dw}{dr}(\frac{du}{dr}+v\frac{u}{r}+\frac{1}{2}{(\frac{dw}{dr})}^{2})=\frac{1}{r}\int_{0}^{r} qrdr$$

Where $D$ is the bending modulus, $E_{2D}$ is the 2D Young’s modulus, q is the internal pressure. The bending modulus here is not directly related to the in-plane Young’s modulus and Poisson’s ratio, which is fundamentally different from that in classical plate theory. This equation balances the internal moments generated by the bending stiffness and the contribution of in-plane membrane force against the applied lateral loading.

To render the problem dimensionless and enhance numerical stability, all quantities are scaled by the effective thickness:

$$h_{e}=\sqrt{\frac{12(1-v^{2})D}{E_{2D}}}$$

The dimensionless variables are defined as:

$\bar{r}=\frac{r}{h_{e}}$*,* $\bar{u}=\frac{u}{h_{e}}$*,* $\bar{w}=\frac{w}{h_{e}}$*,* $\bar{q}=\frac{q{h_{e}}^{3}}{D}$

In addition, the deflection $w$ is replaced with the angle of rotation, $\theta(r)=dw/dr$. The coupled governing equations for the in-plane displacement $u(\bar{r})$ and $\theta(\bar{r})$ can be written as:

$$g(\bar{r})=\frac{d^{2} \bar{u}}{d\bar{r}^{2}}+\frac{1}{\bar{r}}\frac{d \bar{u}}{d\bar{r}}-\frac{\bar{u}}{\bar{r}^{2}}+\frac{1-v}{2\bar{r}}\theta^{2}+\theta\frac{d\theta}{d\bar{r}}=0$$

This equation represents the in-plane force balance in dimensionless form. The first three terms correspond to the membrane’s radial stretching response, while the last two terms represent geometric nonlinearities arising from the coupling between in-plane and out-of-plane deformation.

The out-of-plane force balance in dimensionless form is:

$$f(\bar{r})=\frac{d^{2}\theta}{d\bar{r}^{2}}+\frac{1}{\bar{r}}\frac{d\theta}{d\bar{r}}-\frac{\theta}{\bar{r}^{2}}-12\theta(\frac{d\bar{u}}{d\bar{r}}+v\frac{\bar{u}}{\bar{r}}+\frac{\theta^{2}}{2})-\frac{1}{\bar{r}}\int_{0}^{\bar{r}} \bar{q}(\bar{r})\bar{r}d\bar{r}=0$$

Here, the first three terms describe bending resistance, the fourth term accounts for the nonlinear coupling between stretching and bending, and the final term represents the total external loading per unit length per unit circumference, including contributions from uniform pressure and indentation. Indentation is modeled as a spherical punch profile:

$$\delta\left( r \right)=\left\{ \begin{aligned} coeff\cdot\sqrt{{R_{indent}}^{2}-r^{2}}, r<R_{indent} \\ 0, r\geq R_{indent} \end{aligned} \right.$$

Where $R_{indent}$ is the contact radius, and $coeff$ serves as a scaling factor that modulates the indentation depth based on the geometry of the indentation profile. This indentation profile modifies the effect load distribution in the out-of-plane equation. Then the effective load is defined as the sum of the contributions from the applied uniform pressure and the indentation effect. It is expressed as:

$$\bar{q}_{eff}(\bar{r})=\bar{q}_{pressure}-\bar{q}_{intent}=\bar{q}-\bar{q}\delta(\bar{r})$$

Therefore, the tip-loading force at the center position of the probe (*r* = 0 nm) is:

$$f=\bar{q}_{intent}\times\pi{R_{indent}}^{2}$$

We have simulated the bubble profile to match the aspect ratio (h/*r* ≈ 0.2) of the bubble investigated in our study, as shown in **Fig. S6.1**. Subsequently, we calculated the deformation profiles and strain distributions resulting from the tip strain. The ratio between the contact area for the tip loading and the bubble radius is set as 0.09 to match the estimated ratio between the tip curvature and the bubble radius. In the modeling, we calculated both radial and circumferential strain distributions, which are in-plane strain in polar form. As shown in **Fig. S6.2**, the as-formed bubble exhibits a strain gradient that decreases from the bubble apex. When the indentation force is applied to the bubble region, it causes inward bending of the membrane (**Fig. S6.2a**), with the maximum deflection occurring at the force-loading area. Simultaneously, the tip-loading force induces a strong strain field in the indented area, where the maximum strain is located directly under the tip area of contact. These simulated strain profiles are produced by a force value of 65 nN at the center position of the probe, using a coefficient of 30. In comparison, the simulated deformation in the *z*-direction in **Fig. S6.2a**, corresponds to our experiment when the tip indents around 25 nm into the bubble. The calculated strain is increased by about 65%. By using a gauge factor of 100 meV/%, the expected emission energy shift is 165 meV, which is close to the value of approximately 162 meV that we observed experimentally.

Notably, our model makes some simplifications: 1. We treated the edge of the bubble as clamped, similar to the approach taken by [Adv. Mater. **33**, 2008234, 2021]; 2. We do not consider the van der Waals force between substrate and 2D material. [J. Appl. Mechanics, **80**, 040905, 2013] has shown that the impact of the van de Waals force is small under high pressure; 3. We do not account for how components trapped within the bubble might influence the deformation during the nanoindentation. Nevertheless, our simulation captures the primary effect induced by the tip and provides a direct visualization of the deformation and strain profile under the tip indentation.


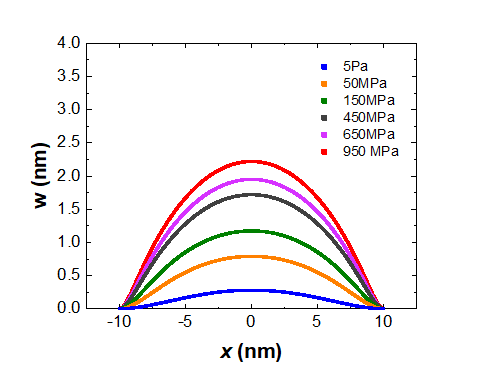


Fig. S6.1 Comparison of the defection profiles for a WSe_2_ monolayer bubble under different internal pressure using the following parameters: $E_{2D}$ is 258 nN/nm [ACS nano, **15**, 2600 (2021)], $v$ is 0.19 [Appl. Phys. Lett. **102**, 012111 (2013)], D is 1.7925 nN$\times$nm [Nanotechnology, **31**, 43LT01, (2020)], and the bubble radius is 10 nm.


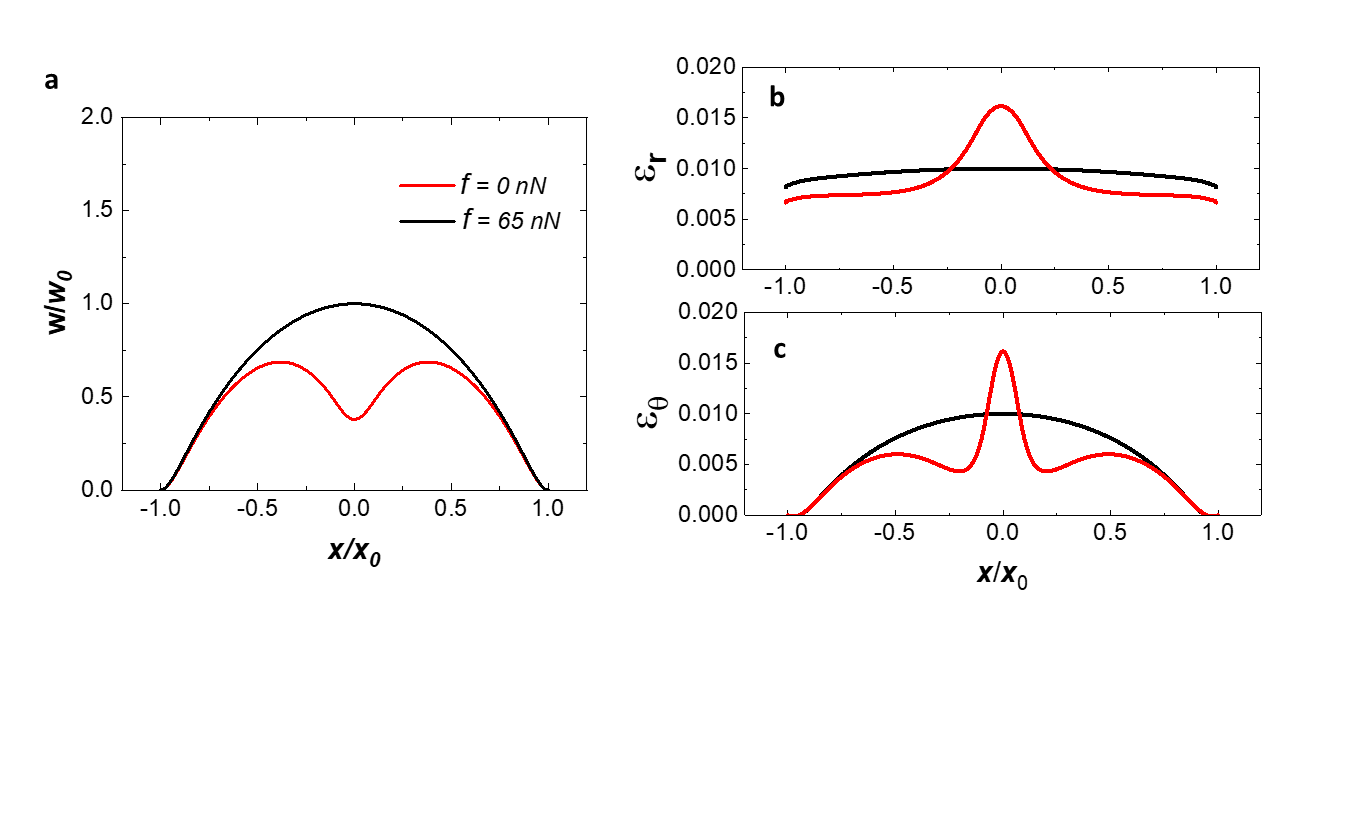


Fig. S6.2: Calculation of deflection and strain profiles of the bubble under indentation load. (b) Comparison of the deflection profiles for a WSe_2_ monolayer bubble with and without indentation load, where the ratio between the radius of the tip-contact area and the bubble radius is 0.9, a tip-loading force *f* = 65 nN is applied at the center position of the probe. Comparison of the radial (c) and circumferential (d) strain distributions for a WSe_2_ monolayer bubble with and without indentation load. Red curves indicate cases under tip load. All the strain value is divided by a constant of 3.541 to match the maximum strain value of the bubble (*f* = 0 nN) with the strain value derived from the AFM heigh map in Fig. S1.

**Note S7: Evolution of bubble emission as the excitation power increases**


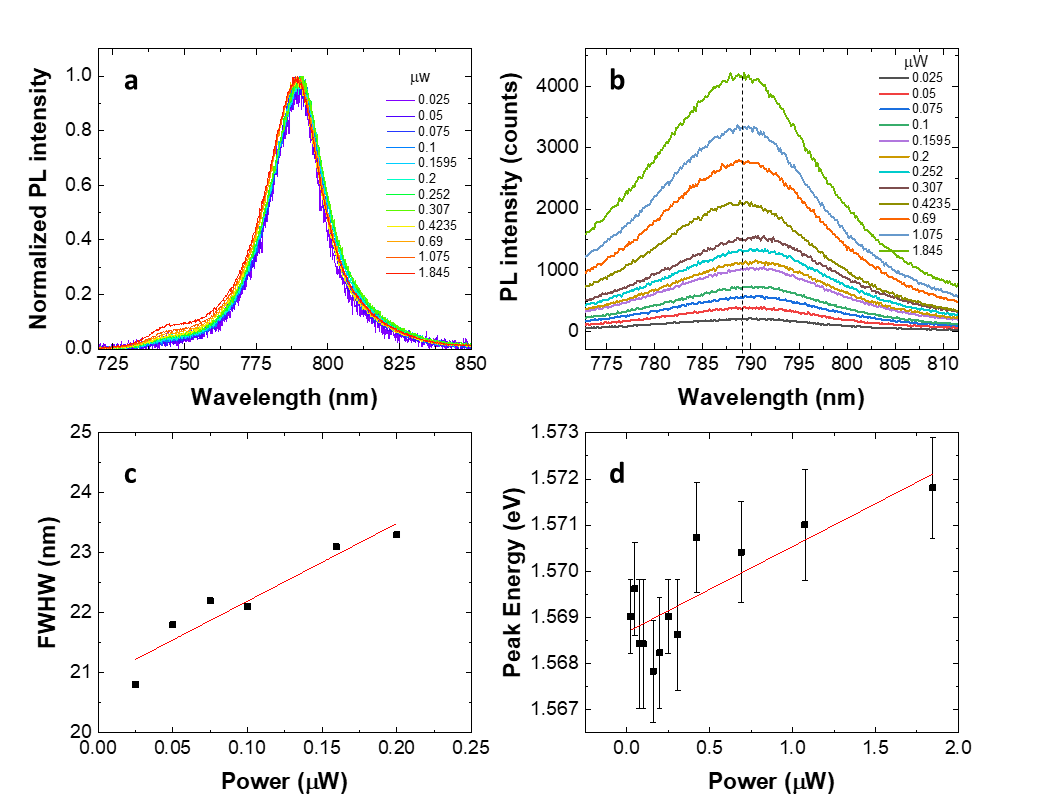


Fig. S7: PL spectra of the bubble emission peak as a function of excitation power. (a) Normalized spectra. (b) Zoomed-in bubble emission peak, the dashed line is at around 789 nm is for guidance purposes. (c) Linewidths broaden within the low excitation power range, where the spectra can be fitted with a single Lorentzian peak. (d) Extracted peak wavelengths blueshift as the excitation power increase.
